# Supplementary material for: A whole-animal phenotypic drug screen identifies suppressors of atherogenic lipoproteins
Source: eLife. 2026 Aug 27;14:RP105314. doi: 10.7554/eLife.105314 (PMC13521566; doi:10.7554/eLife.105314)
Supplement: Supplementary file 2 [file elife-105314-code1.zip › JHDL_validation_result/Riboflavine tetrabutyrate_DJK20210124_01.pdf]

Validation of Riboflavine tetrabutyrate

6 total outliers removed

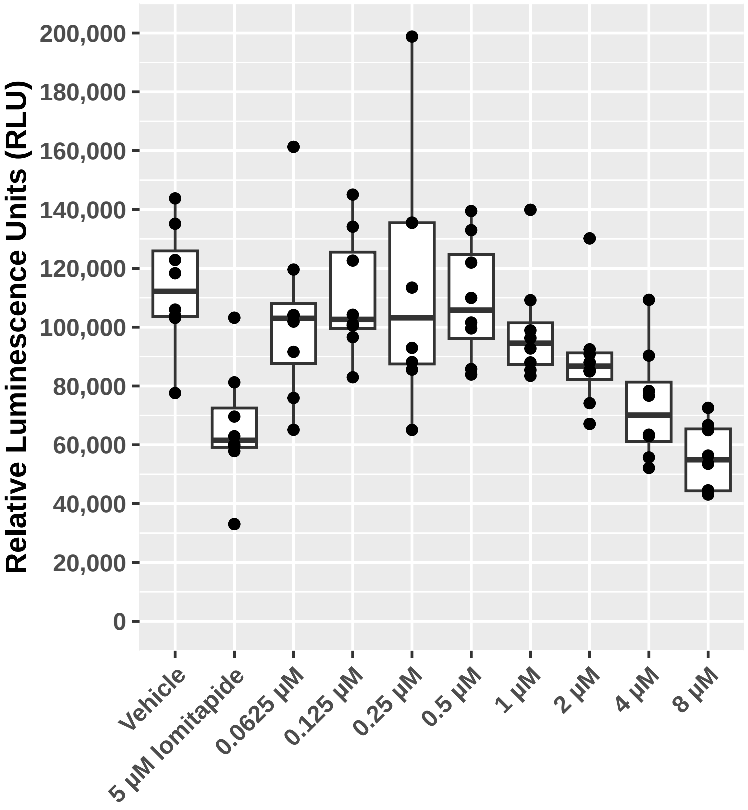

Experiment: DJK20210124_01
